# Serums miR-24-3p and miR-1301-3p as Potential Biomarkers in MEN1 Syndrome

**DOI:** 10.3390/ijms26115076

**Published:** 2025-05-24

**Authors:** Simone Donati, Cinzia Aurilia, Francesca Marini, Francesca Giusti, Gaia Palmini, Irene Falsetti, Federica Cioppi, Francesco Ranaldi, Teresa Iantomasi, Arcangelo Moro, Francesco Tonelli, Maria Luisa Brandi

**Affiliations:** 1 Department of Experimental and Clinical Biomedical Sciences, University of Florence, Viale Pieraccini 6, 50139 Florence, Italy; simone.donati@unifi.it (S.D.); cinzia.aurilia@unifi.it (C.A.); francesca.giusti@unifi.it (F.G.); irene.falsetti@unifi.it (I.F.); francesco.ranaldi@unifi.it (F.R.); teresa.iantomasi@unifi.it (T.I.); 2 FirmoLab, Fondazione F.I.R.M.O. Onlus and Stabilimento Chimico Farmaceutico Militare (SCFM), 50141 Florence, Italy; francesca.marini@fondazionefirmo.com (F.M.); gaia@fondazionefirmo.com (G.P.); francesco.tonelli@unifi.it (F.T.); 3 Metabolic Bone Diseases Unit, University Hospital of Florence, AOU Careggi, 50139 Florence, Italy; cioppif@aou-careggi.toscana.it; 4 Stabilimento Chimico Farmaceutico Militare (SCFM)—Agenzia Industrie Difesa (AID), 50141 Florence, Italy; arcangelo.moro@aid.difesa.it

**Keywords:** multiple endocrine neoplasia type 1, epigenetics, non-coding RNAs, circulating microRNAs, neuroendocrine tumors, biomarkers

## Abstract

Multiple endocrine neoplasia type 1 (MEN1) is a rare hereditary tumor syndrome caused by inactivating mutations of the *MEN1* gene and characterized by the occurrence of multiple endocrine tumors within a single patient (i.e., parathyroid, pituitary, and pancreatic neuroendocrine tumors (NETs)). However, the lack of a genotype–phenotype correlation does not allow individual disease evolution to be foreseen. Epigenetic factors, such as microRNAs, are suspected to contribute to MEN1 tumorigenesis, presumably explaining the lack of genotype–phenotype association. Our previous studies indicated miR-24-3p, miR-1301-3p, miR-664a-3p, and miR-4258 as potentially involved in MEN1 parathyroid tumorigenesis. In this study, we examined the expression of two circulating microRNAs (c-miRNAs), miR-24-3p and miR-1301-3p, in the serum of MEN1 patients. c-miRNAs were evaluated by RT-qPCR in serum collected from 25 MEN1 patients and 25 age- and gender-matched healthy volunteers (HCs). Receiver operating characteristic (ROC) curves were constructed to determine miRNA sensitivity and specificity. RT-PCR analysis revealed that expression levels of circulating miR-1301-3p were significantly downregulated, while those of miR-24-3p were significantly upregulated in the serum of MEN1 patients compared to HCs. Additionally, ROC analysis exhibited a good diagnostic power for both miRNAs (area under the ROC curve (AUC) values: 0.7356 and 0.7928 for miR-1301-3p and miR-24-3p, respectively) in distinguishing MEN1 patients from matched HCs. These preliminary data suggest circulating miR-1301-3p and miR-24-3p as potential non-invasive diagnostic biomarkers for MEN1 syndrome, regardless of different clinical phenotypes and *MEN1* mutation types.

## 1. Introduction

Multiple endocrine neoplasia type 1 (MEN1) (OMIM #131100) is a rare genetic neoplastic syndrome, characterized by a multiple onset of endocrine and non-endocrine tumors in a single patient. Suspicion of MEN1 arises when a patient presents two or more of the classical clinical features associated with the syndrome, such as the development of tumors in the glands belonging to the so-called “P triad” (i.e., parathyroids, pancreas, and pituitary) [1]. To date, various combinations of about 20 different types of endocrine and non-endocrine tumors have been found in MEN1 patients [2].

MEN1 is caused by germline heterozygous loss-of-function mutations in the *MEN1* tumor suppressor gene at locus 11q13, which encodes the oncosuppressor protein menin, whose reduced or totally absent activity is responsible for multiple tumor development [3,4]. The inheritance of MEN1 is autosomal dominant, with a penetrance greater than 50% by the age of 20 and complete by the age of 50 years [5]. Neuroendocrine tumors in MEN1 show biallelic loss of the *MEN1* gene; loss of the first allele occurs at the germinal level (heterozygosity state), followed by loss of the second *MEN1* allele at the somatic level (loss of heterozygosity; LOH), in target neuroendocrine cells, according to the Knudson’s “two-hit” hypothesis for conventional tumor suppressor genes [6,7].

Despite the fact that more than 1500 different somatic and germinal mutations of the *MEN1* gene have been described so far [6,8], no reliable direct correlation between MEN1 genotype and clinical phenotype is available, thus hampering the possibility of foreseeing MEN1 individual clinical manifestations and, therefore, not allowing the development of a personalized diagnostic and therapeutic approach to the disease.

Given the fact that the clinical phenotype in MEN1 differs within members of the same family and even in homozygous twins [9], individual manifestations of the syndrome are suspected to be the result of interaction between the genetic defect and exogenous influences that modulate the activity of epigenetic factors, such as DNA methylation, histone modification, and microRNAs (miRNAs). These epigenetic mechanisms may, indeed, act as cofactors of the genetic variant in contributing to the onset of different tumors in patients bearing the same *MEN1* mutation [9]. miRNAs are small non-coding RNAs (18–25 nucleotides) that negatively regulate gene expression at the post-transcriptional level [10] by targeting the 3’ untranslated region (3’-UTR) of specific RNA messengers (mRNAs) and, thus, preventing protein translation [11,12,13]. Deregulation of miRNA expression and/or activity has been found in various human tumors [14], prompting the possibility of using these molecules as possible diagnostic, prognostic, and therapeutic biomarkers in cancer.

A role for microRNAs in MEN1-associated tumor initiation and development has been documented.

Previous studies have demonstrated a “negative feedback loop” between *MEN1*, miR-24-3p, and menin, showing this miRNA to directly target the 3’UTR of *MEN1* mRNA and to inhibit the expression of menin, mimicking the second hit of Knudson’s tumorigenesis model, in a still reversible manner, before irreversible genetic *MEN1* somatic loss [15,16]. Three more miRNAs (miR-664a-3p, miR-1301-3p, and miR-4258) may have a possible role in the biology of MEN1 parathyroid neoplasia by targeting oncosuppressor genes known or suspected to be involved in the development of familial forms of parathyroid tumors, such as *CDC73*, *CDKN1B*, *CDKN2C*, *RET*, *AP2S1*, *CCND1*, *CCDN2*, and *CTNNB1* [17].

In addition to their intracellular localization, the presence of extracellular miRNAs was found in a variety of biological fluids [18,19,20,21], where they retain a high stability, probably due to their binding to specific proteins, such as nucleophosmin 1, high-density lipoproteins, and Argonaute 2, or due to their inclusion in exosomes, microvesicles, and apoptotic bodies [22,23,24]. The findings reported in four independent studies [25,26,27,28] showed different expression profiles of serum miRNAs (circulating miRNAs; c-miRNAs) between physiological and pathological states. Expression patterns of c-miRNAs in the serum could, thus, directly reflect the physiopathological status of an organism, suggesting that measurement of these molecules may represent a useful non-invasive biomarker to diagnose or follow up the progression of many diseases, including inherited forms of tumors.

In the present study, we aimed to assess whether serum levels of two c-miRNAs, miR-24-3p and miR-1301-3p, differed between MEN1 patients and non-MEN1 healthy controls (HCs).

## 2. Results

### 2.1. Patient Characteristics

The study included 25 MEN1 patients [17 (68%) females and 8 (32%) males] and 25 HCs [13 (52%) females and 12 (48%) males]. The mean age was 44.4 ± 12.12 years (range 14–70) for the MEN1 group and 40.1 ± 10.25 years (range 27–65) for the HC group at the time of inclusion in this study.

Comparison between the 25 MEN1 patients and the 25 healthy controls showed no significant differences in terms of age and gender distribution (*p*-values > 0.05), as shown in Table 1.

The clinical characteristics of the 25 MEN1 patients at the time of blood sampling are shown in Table 2.

After the assessment of the hemolysis degree, three serum samples, one from a MEN1 case (n. 22) and two from controls (XXIII and XXV), were considered hemolyzed and, thus, excluded from the c-miRNA expression analysis.

### 2.2. Selection of a Suitable Endogenous Reference miRNA

**Figure 1:**
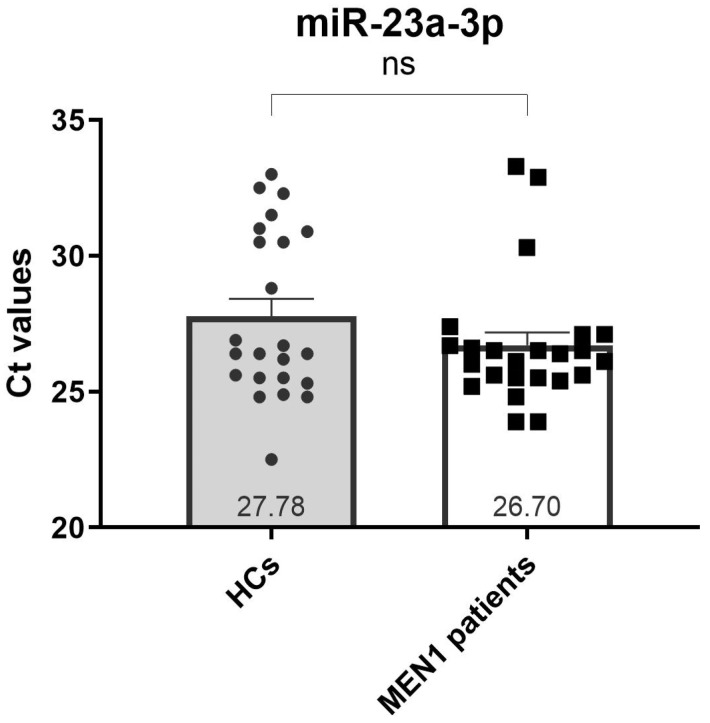
Expression differences of miR-23a-3p between MEN1 patients and HCs. Data are expressed as mean values ± standard error (SE).

Our analysis revealed that miR-23a-3p met all the selection criteria for being considered a reliable reference miRNA, with an average SE of 0.40 (Table 3). Furthermore, no significant differences were detected between MEN1 patients and HCs in the mean expression of miR-23a-3p (Figure 1). On the contrary, expressions of miR-93-5p and miR-191-5p were not detected in all samples, making these two miRNAs not eligible as endogenous reference miRNAs for expression data normalization.

### 2.3. Analysis of c-miRNA Expression Levels and Assessment of the Diagnostic Value of miR-1301-3p and miR-24-3p

Hsa-miR-1301 is a newly discovered miRNA encoded by a gene located within chromosome 2p23.3 [29]. The hsa-miR-1301 precursor can produce two mature miRNA products, miR-1301-3p and miR-1301-5p. However, all existing miR-1301-related studies only focused on miR-1301-3p, and there have been no studies of miR-1301-5p so far. The miR-1301-3p was selected for the present study since in a previous study by our Research Group, it was found to be differentially regulated in MEN1 parathyroid adenomas with LOH at 11q13 locus compared to the non-LOH counterpart and healthy parathyroid tissue [17]. In silico analysis with the ComiR tool revealed that miR-1301-3p may potentially target genes involved in parathyroid tumorigenesis, such as *RET* oncogene (responsible for the MEN2A syndrome), the *Cyclin-Dependent Kinase Inhibitor 1B* (*CDKN1B*) tumor suppressor gene (responsible for the MEN4 syndrome), *RB Transcriptional Corepressor 1* (*RB1*) tumor suppressor gene, *vitamin D receptor* (*VDR*) gene, and *PR/SET Domain 2* (*PRDM2*) gene [17].

Hsa-miR-24-3p is encoded by two separated genomic loci: one gene cluster localized on chromosome 9q22, which includes miR-23b, miR-24-1-3p and miR-27b, and the other located on chromosome 19p13, which comprises miR-23a, miR-24-2, and miR-27a. The miR-24-3p was selected for the present study, since previous studies revealed the existence of a direct negative feedback loop between the miR-24-3p, *MEN1* mRNA, and menin protein, both in parathyroid tumor tissues from MEN1 patients and in endocrine pancreas-derived cell lines (the MIN6 mouse insulinoma cells, the βlox5 immortalized human pancreatic beta cells, and the BON1 human cell line derived from a lymph node metastasis of a pancreas neuroendocrine tumor) [15,16,30,31]. Previous studies revealed that miR-24-3p was implicated in the negative regulation of *CDKN1B* and *CDKN2C* genes, which encode, respectively, the cell cycle inhibitors p27^kip1^ and p18^ink4c^, through the suppression of menin translation [16,32].

Two additional miRNAs, miR-4258 and miR-664a-3p, that had been found to be significantly differentially expressed in LOH *MEN1* parathyroid adenomas vs. non-LOH parathyroid adenomas [17] were excluded from the present study, since a preliminary screening of a pool of serum samples from MEN1 patients and a pool of serum samples from HCs performed in our laboratory showed no significant difference in the circulating levels of these two miRNAs between the two groups.

**Figure 2:**
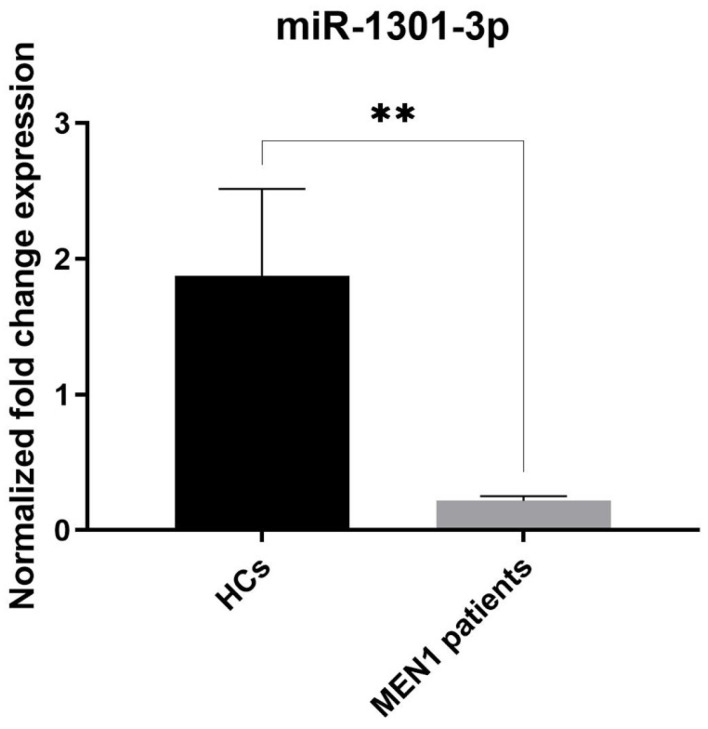
Serum expression levels of miR-1301-3p in MEN1 patients compared to the HC group. miR-1301-3p was significantly lower in MEN1 cases (** = *p*-value < 0.01). Data are expressed as mean values ± standard error (SE).

**Figure 3:**
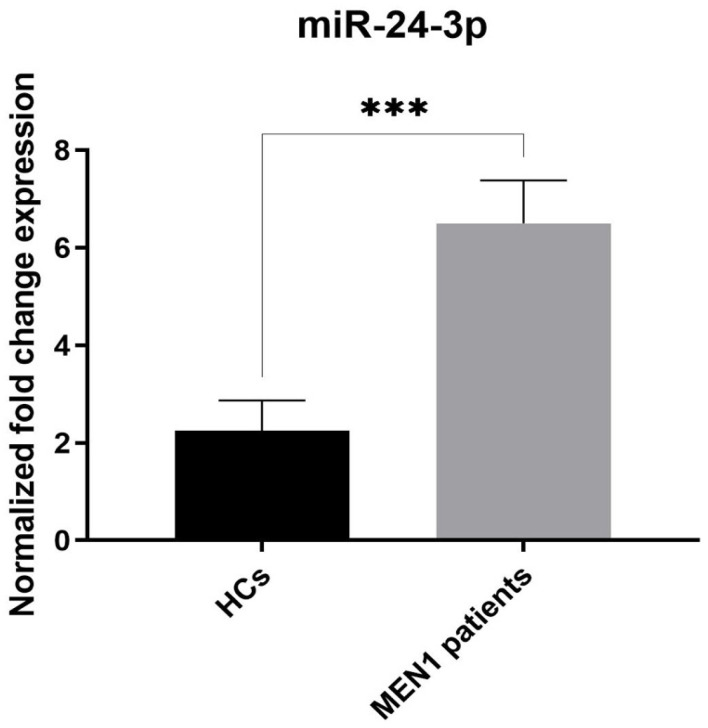
Serum expression levels of miR-24-3p in MEN1 patients compared to the HCs group. miR-24-3p was significantly higher in MEN1 cases (*** = *p*-value < 0.001). Data are expressed as mean values ± standard error (SE).

Expression levels of miR-24-3p and miR-1301-3p in serum samples of the MEN1 patient group (*n* = 24) and HCs group (*n* = 23) were analyzed by qPCR. It was observed that expression levels of miR-1301-3p were significantly decreased in the MEN1 cohort compared with the HC cohort (Figure 2, *p*-value < 0.05). Meanwhile, qPCR showed that serum miR-24-3p levels were significantly upregulated in MEN1 patients compared to healthy subjects (Figure 3, *p*-value < 0.05).

**Figure 4:**
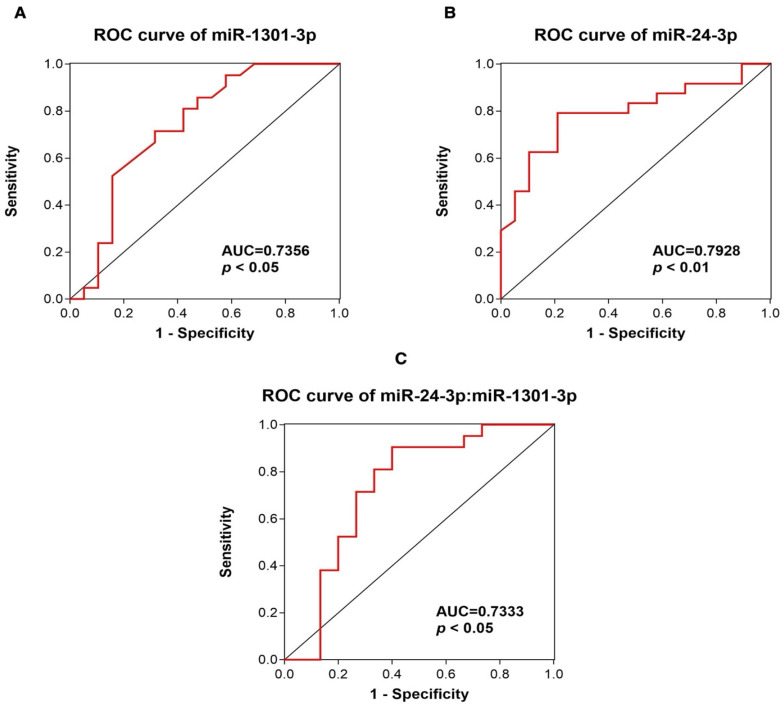
Analysis of the diagnostic value of miR-1301-3p, miR-24-3p, and the combination of miR-24-3p and miR-1301-3p. (**A**) The ROC curve of miR-1301-3p (red line) exhibited a good diagnostic power for distinguishing the MEN1 patient group vs. HC group, with an average AUC of 0.7356, with 68% specificity and 71% sensitivity when the best cut-off value was applied. (**B**) The ROC curve of miR-24-3p (red line) exhibited a good diagnostic power for distinguishing the MEN1 patient group vs. HC group, with an average AUC of 0.7928, with 79% specificity and 79% sensitivity when the best cut-off value was applied. (**C**) The ROC curve of miR-24-3p:miR-1301-3p (red line) exhibited a good diagnostic power for distinguishing the MEN1 patient group vs. HC group, even though it exhibited a lower diagnostic value compared to each single microRNA, with an average AUC of 0.7333, with 60% specificity and 90% sensitivity when the best cut-off value was applied.

We carried out a ROC curve analysis to evaluate the diagnostic value of miR-1301-3p and miR-24-3p in discriminating MEN1 patients from HCs. As shown in Figure 4, ROC analysis revealed that the AUC for miR-1301-3p was 0.7356 (95% Confidence Interval (CI) 0.5729–0.8983)), with 71% sensitivity and 68% specificity when the best cutoff value was applied (<0.2485), and for miR-24-3p was 0.7928 (95% CI 0.6561–0.9294), with 79% sensitivity and 79% specificity when the best cutoff value was applied (>3.803), indicating these serum miRNAs as promising diagnostic biomarkers for MEN1 syndrome (Figure 4A,B). The combination of the two miRNAs (miR-24-3p:miR-1301-3p) resulted in a reduced discriminatory capability (AUC of 0.7333, 90% sensitivity and 60% specificity 76.2% when the best cutoff value was applied (>5.684) (*p*-value < 0.05; 95% CI 0.5470 to 0.9197)) compared to the expression of the two circulating miRNAs alone (Figure 4C).

## 3. Discussion

A direct role of miRNA dysregulation in human cancers, including MEN1 tumorigenesis, has been reported. Since the expression of miRNAs is tissue-specific, different tumors usually have distinctive intracellular miRNA expression profiles. Selected miRNAs can be released, through exosomes, by tumor cells, being characteristics of a specific tumor microenvironment and, ultimately, representing the underlying mechanism of the tumor-specificity of c-miRNAs. Different serum levels of c-miRNAs were correlated with the degree of tumor progression, and the presence of specific c-miRNAs was associated with cancer development, adjacent tissue invasion, and distant metastasis [33]. Therefore, c-miRNAs have become new candidates as non-invasive and tumor-specific diagnostic and prognostic biomarkers in a variety of human neoplasms.

The management and identification of multiple NET subtypes may benefit from the use of a neuroendocrine neoplasms test (NETest). The latter is a standardized blood biomarker assay composed of 51 transcripts found to be upregulated in NETs. In comparison with other available single-secreted NET biomarker tests, such as chromogranin A (CgA), NETest is considered a more accurate biomarker for imaging, grade, disease status and progression, and response to therapies [34,35]. Despite this tool being designed for sporadic NETs, the utility of the NETest has also been demonstrated for the detection of multiple NET subtypes in MEN1 individuals. However, as MEN1 patients commonly have multiple concomitant tumors, there is likely to be a need to make adjustments to the existing NETest to improve the ability to diagnose such patients.

The research carried out by Soldevilla and colleagues [36] assessed the potential of using miRNAs as prognostic biomarkers in NETs, discovering a distinctive pattern of eight miRNAs (i.e., miR-17-5p, miR-18-5p, miR-19a-3p, miR-20a-5p, miR-20b-5p, miR-92a-3p, miR-203a-3p, and miR-210-3p) capable of predicting the survival of patients with GEP and lung NETs across three prognostic groups (5-year survival rates of 80%, 66%, and 36%), additionally pinpointing genes and regulatory mechanisms associated with the prognosis of NET patients.

Recently, two studies investigated c-miRNA expression in MEN1, identifying specific c-miRNAs differentially expressed in the serum or plasma of MEN1 patients with respect to a phenocopy of the syndrome and/or HCs [37,38]. The identification of specific c-miRNA signatures associated with MEN1 syndrome and/or with different clinical phenotypes could help the diagnostic management of MEN1 syndrome and MEN1 tumors, in combination with *MEN1* genetic testing and with the currently used clinical, biochemical, and radiological approaches. Moreover, since miRNA expression is influenced and rapidly modified by changes in endogenous and exogenous conditions, periodical evaluation of changes in specific c-miRNA signatures could be useful to monitor the progression of MEN1 tumors and/or the response to therapies.

Here, we selectively evaluated, for the first time, whether serum levels of two specific miRNAs, miR-24-3p and miR-1301-3p, differed between MEN1 patients and non-MEN1 HCs. miR-24-3p was selected, since it had been previously demonstrated to directly interact with *MEN1* mRNA and to negatively regulate the expression of menin protein, both in parathyroid tissues from MEN1 patients and endocrine pancreas cells [15,16,30]. miR-1301-3p was selected since it had been demonstrated to be upregulated in LOH *MEN1* parathyroid adenomas, both with respect to non-LOH *MEN1* parathyroid adenomas and control tissue [17].

In the analysis of serum levels of c-miRNAs, the first challenge is to select a suitable reference miRNA to be used to correctly normalize expression data. Indeed, although the qPCR method is widely used for c-miRNA expression analysis, no consensus exists about which are the best endogenous reference miRNAs to choose for normalizing extracellular miRNA expression levels [39]. Here, we tested and identified, for the first time, an endogenous miRNA, miR-23a-3p, that resulted to be a good reference miRNA for normalization of serum miRNA concentration in the MEN1 syndrome, being equally expressed both in patients and HCs. Our result is consistent with previous data from Blondal et al. [40], who found miR-23a-3p to be a relatively stable miRNA in plasma and serum, and whose expression levels were not affected by hemolysis.

Then, using miR-23a-3p as a reference circulating miRNA, we measured the expression of miR-1301-3p and miR-24-3p in the serum of MEN1 patients versus healthy individuals, finding the first was significantly less expressed and the second significantly more expressed in MEN1 patients than in controls.

The miR-1301-3p has been reported to be abnormally expressed in 14 types of tumors, being downregulated in 11 of them, such as oral squamous cell carcinoma [41], cervical cancer [42], esophageal cancer [43,44,45], laryngeal squamous cell carcinoma [46], papillary thyroid carcinoma [47,48,49], glioma [29,50,51], chronic myeloid leukemia [52], clear cell renal cell carcinoma [53], bladder cancer [54], osteosarcoma [55,56,57], and colorectal cancer (CRC) [58,59,60]. In the field of MEN1, Luzi et al. [17] showed that miR-1301-3p was significantly increased in MEN1 parathyroid adenomas with LOH at 11q13 locus compared to the non-LOH counterpart and a healthy parathyroid control pool. In apparent contrast to what was observed in the aforementioned study [17] regarding the upregulation of miR-1301-3p in MEN1 parathyroid adenomas with 11q13 LOH, here we found that miR-1301-3p expression levels were significantly reduced in the serum of MEN1 patients vs. HCs. This apparent discrepancy is in line with data previously published in the literature and reporting that expression levels of various miRNAs often show an opposite trend between the serum and tumor tissue of oncological patients [61,62,63,64,65]. These differences may be caused by the complexity of the biological regulation of miRNA expression and the fact that miRNA changes in the blood may not only derive from intrinsic changes within tumor cells and their release into the circulation, but may also be altered due to the host immune response or inflammatory reactions, as well as by the fact that, in the case of complex diseases, such as MEN1, in which different and multiple tumors may occur in a single patient, levels of specific circulating miRNAs can be the result of these multiple tumors rather than of a single pathology. Despite finding in this pioneering study that miR-1301-3p was significantly lower in the serum of MEN1 patients, further studies are needed to confirm a possible diagnostic feature of this circulating miRNA in MEN1 patients.

A study by Luzi et al. [15] hypothesized the existence of an autoregulatory mechanism involving miR-24-3p, *MEN1* mRNA, and menin, which appears to mimic the second Knudson’s hit in tissues in which *MEN1* LOH has not yet occurred, thereby triggering the onset of parathyroid hyperplasia/adenoma, in an epigenetic and still reversible manner, before the irreversible genetic *MEN1* LOH occurs. This mechanism could be suspected to initiate hyperplastic changes in parathyroid chief cells, progressively evolving to neoplasia, and it could also be postulated for pancreatic, duodenal, and other MEN1-related neuroendocrine neoplasms [66]. The differential expression of miR-24-3p in biofluids as a non-invasive diagnostic biomarker has been considered in patients with hepatocellular carcinoma (HCC) [67,68]. Serum levels of miR-24-3p could successfully discriminate HCC patients from those with chronic liver disease. In addition, its expression was associated with HCC patient survival; miR-24-3p over-expression was, indeed, correlated with a poor prognostic factor for overall survival and disease-free survival of hepatitis B virus-related HCC patients [68]. Interestingly, the association between serum miR-24 expression and the risk of relapse in breast cancer (BC) patients was also investigated, finding that the overexpression of this miRNA, as well as of miR-155, was highly predictive of early BC relapse [69]. Similarly to what was observed in these studies, we found higher levels of miR-24-3p in the serum of MEN1 patients compared to HCs, as a possible confirmation of the oncogenic nature of miR-24-3p. Moreover, in a study by Yavropoulou et al., miR-24-3p expression was found to be significantly increased, at the tissue level, in sporadic parathyroid adenomas compared to normal parathyroid tissues, even though no significant differences were found in the serum of patients with sporadic parathyroid tumors compared to healthy controls (Fold change > 100) [70]. In the study, the authors found that the expression of miR-24-3p inversely correlated with the expression of the *MEN1* gene in tumor samples, suggesting that the epigenetic silencing of the *MEN1* gene by miR-24-3p in parathyroid adenoma may occur regardless of whether a patient is affected or not by MEN1 syndrome. In this light, the overexpression of miR-24-3p could represent a dysregulated epigenetic mechanism leading to primary hyperparathyroidism (PHPT) due to menin expression loss in the parathyroid gland(s), both in the MEN1 syndrome and in sporadic counterparts. However, not all patients analyzed in our study had developed PHPT at the time of blood sampling, making us speculate that the increased circulating miR-24-3p may have been due to a still undiagnosed PHPT or to the presence of other MEN1 neuroendocrine tumors. The significant difference in serum values of miR-24-3p between MEN1 patients and healthy subjects seems to indicate that this circulating miRNA as a possible additional biochemical marker in the diagnosis of MEN1 syndrome. Another study by Hwang et al. [71] was specifically carried out to identify a tissue miRNA signature for discriminating sporadic from hereditary parathyroid tumors. They found that miR-199b-5p expression levels were significantly decreased and negatively correlated with parathyroid hormone levels in sporadic parathyroid adenomas and upregulated in the inherited counterpart. However, no previous studies have evaluated its circulating levels in the MEN1 population.

ROC analysis exhibited a good diagnostic power for both miRNAs (AUC values: 0.7356 and 0.7928 for miR-1301-3p and miR-24-3p, respectively) in differentiating MEN1 patients from matched HCs. These preliminary data, which need to be replicated and validated in additional analyses, indicate that the expression of these two c-miRNAs is associated with MEN1 syndrome, regardless of the different clinical phenotypes and *MEN1* mutation types, and it may have diagnostic potential for this syndrome.

However, our study presents some limitations.

Our MEN1 patients exhibited a high variety of clinical phenotypes, ranging from an asymptomatic young case to variable combinations of one to six different endocrine and non-endocrine tumors. Therefore, the limited number of MEN1 patients analyzed (*n* = 24) did not allow us to evaluate whether there was a correlation between miR-24-3p and miR-1301-3p serum levels and different clinical phenotypes. Evaluations of serum miRNA on larger and independent cohorts of MEN1 patients, also with respect to different clinical phenotypes, are therefore needed.

Moreover, our study did not take into account ongoing treatments that could have interfered with the extracellular miRNAs released by tumor cells. It would be interesting to periodically evaluate levels of serum miRNAs in *MEN1* mutation carriers who have not yet developed any signs or symptoms of MEN1 syndrome and are not undergoing any medical treatment, to monitor how c-miRNA levels can vary with the progression of the disease and whether they could be early indicators of tumor occurrence.

In conclusion, growing evidence is emerging for the effectiveness of analyses of serum c-miRNA levels as non-invasive and easily measurable biomarkers, which, in addition to the pre-existing instrumental and biochemical approaches, could help clinicians to provide an earlier and more accurate diagnosis of several human tumors, both as sporadic forms or in the context of inherited non-syndromic and syndromic neoplasia. However, the few currently available data on c-miRNA signatures in MEN1 patients, including those found in this study, are insufficient and not sufficiently clear to define the possible role these biochemical parameters may have in the diagnostic management of the syndrome; further and targeted studies are indeed required to try to solve this issue and to translate the use of serum miRNA measurement into clinical practice.

## 4. Materials and Methods

### 4.1. Patient Information and Serum Collection

This study protocol was approved by the Ethical Committee of the Azienda Ospedaliero-Universitaria Careggi (AOUC), Florence, Italy (Rif. CEAVC BIO 16.018). A written informed consent was obtained from each participant. All procedures were conducted in accordance with the Helsinki Declaration and its later amendments. Twenty-five MEN1 patients and 25 age- and gender-matched HCs were recruited for the study in 2018–2019. HCs were selected among people without a history of benign or malignant tumors and with a reported apparent good health status, not taking any chronic medications at the time of the blood sampling or in the previous 12 months. Samples of five milliliters of peripheral blood were collected from 25 patients with proven MEN1 syndrome during health checkups at the AOUC Hospital (coded as Arabian numbers 1–25) and 25 age- and gender-matched HCs (coded as Roman numbers I–XXV) under fasting conditions via venipuncture into BD Vacutainer SSTTM Advance (BD-Belliver Industrial Estate, Plymouth, UK). Each blood sample was processed within 1 h post-collection. After a blood clotting of 20–30 min, serum was recovered via a first centrifugation at 1500× *g* for 20 min at 4 °C, followed by high-speed second centrifugation at 16,000× *g* for 10 min at 4 °C, performed to completely remove possible contaminants and/or any remaining cells. The recovered serum supernatant was aliquoted into RNase-free tubes and immediately stored at −80 °C until analysis.

### 4.2. Hemolysis Assessment

Since hemolysis could notably alter the evaluation of expression levels of miRNAs in the serum samples, the degree of hemolysis was assessed in all the collected samples by using three different methods. First, we assessed hemolysis through a simple visual detection of serum samples for a pink to red discoloration (an indicator of severely hemolyzed serum samples). Second, the degree of hemolysis in serum samples was monitored by measuring the optical density of free hemoglobin levels at 414 nm using NanoDrop^®^ ND-1000 Spectrophotometer (Thermo Scientific, Waltham, MA, USA). Serum samples were considered as being hemolyzed if the absorbance reading at λ = 414 nm exceeded a value of 0.2 [72]. Finally, we determined hemolysis using a miRNA-based approach through the ratio of delta cycle threshold (C_t_) value of a stable serum miRNA (miR-23a-3p), and a red blood cell (RBC)-enriched miRNA (miR-451a) (Table 4), by quantitative polymerase chain reaction (qPCR), using miScript SYBR Green PCR Kit (Qiagen, Hilden, Germany). A ratio of miR-23a-3p to miR-451a (ΔC_t_: (C_t_ miR-23a-3p − C_t_ miR-451a) greater than or equal to 7.5 was an indicator of hemolysis [40].

### 4.3. RNA Extraction

Total RNA, including small RNAs, was extracted from 200 μL of serum, using miRNeasy Serum/Plasma Kit (Qiagen, Hilden, Germany) and miRNeasy Serum/Plasma Spike-In Control (Qiagen, Hilden, Germany), following the manufacturer’s protocol for liquid samples. Briefly, serum samples were thawed and 1 mL of Qiazol lysis reagent was added. The samples were then incubated for 5 min at room temperature (RT). Subsequently, after chloroform addition, the denatured samples were separated into aqueous and organic phases. The aqueous phase containing RNA molecules was recovered and ethanol was added to the supernatant to provide the appropriate conditions for RNA molecules of 18 nucleotides for binding upwards to the silica membrane. The sample was then applied to the RNeasy MinElute (Qiagen, Hilden, Germany), where the total RNA remained attached to the membrane and other contaminants were washed away. Finally, total RNA was eluted in RNase-free water. To monitor RNA purification yields and reverse transcription efficiency, we spiked-in a *Caenorhabditis elegans* miR-39-3p synthetic miRNA mimic (cel-miR-39-3p) (Table 4) to a final concentration of 1 × 10^8^ copies/μL, following the initial denaturation step and prior to the addition of chloroform.

### 4.4. Quantitative Polymerase Chain Reaction (qPCR) Analyses

Reverse transcription was carried out using a miScript II RT Kit (Qiagen, Hilden, Germany) using oligo-dT primers, which have a 3’-degenerate anchor and a 5’-universal tag sequence, allowing amplification of mature miRNA in the qPCR step, in a final volume of 20 μL, according to the manufacturer’s instructions. The 20 μL reverse transcription reaction mixture contained 2 μL 5× miScript HiSpec Buffer, 2 μL 10× miScript Nucleic Mix, 2 μL miScript Reverse Transcription Mix, 10 μL RNase-free water, and 2 μL purified RNA (containing miRNeasy Serum/Plasma Spike-In Control). On an Eppendorf^®^ Mastercycler^®^ Nexus X2 Thermal Cycler (Eppendorf AG, Hamburg, Germany), the reaction mixture was incubated at 37 °C for 60 min and 95 °C for 5 min. The resulting cDNA was diluted in 200 μL RNase-free water and stored at −20 °C until use, according to the manufacturer’s instructions. qPCR was carried out by miScript SYBR^®^ Green PCR Kit (Qiagen, Hilden, Germany) with predesigned miScript Primer Assays (Qiagen, Hilden, Germany) (Table 4) in a final volume of 10 μL on a Rotor-Gene Q72-Well Rotor (Qiagen, Hilden, Germany), following the manufacturer’s instructions. The qPCR reaction mixture consisted of 5 μL 2× QuantiTect SYBR Green PCR Master Mix, 1 μL 10× miScript Universal Primer, 1 μL 10× miScript Primer Assay, 1 μL template cDNA, and 2 μL RNase-free water. The thermal cycling conditions consisted of an initial polymerase activation step at 95 °C for 15 min, followed by 40 cycles of 94 °C for 15 s, 55 °C for 30 s, and 70 °C for 30 s, with a melting curve analysis carried out at the end of each PCR run to verify the non-specific amplification. All qPCR experiments were carried out in triplicate for each miRNA and a non-template control (NTC) was included in each batch of reactions. The C_t_ values were determined using the fixed threshold setting, and those resulting <35 were accepted for further experimentation. A value above a C_t_ ≥ 35 was treated as background.

### 4.5. Selection of Reference miRNA and qPCR Data Normalization

Since it is essential to identify an endogenous suitable reference miRNA for normalizing c-miRNA expression levels to control the occurrence of false positives and false negatives, we tested three miRNAs as possible reference genes (RGs) by analyzing qPCR data through the comparative ΔCt method.

miR-191-5p was selected from the literature, as it is recommended as the most suitable endogenous control for analyzing the miRNA expression profile in studies on patients affected by a variety of pathological conditions, having the most analogies with that of MEN1 syndrome, including colorectal adenocarcinoma patients, hepatitis B and hepatocellular carcinoma [73], and breast cancer [74]. In addition, we tested two commonly used stable serum miRNAs (miR-93-5p and miR-23a-3p) [39]. We used three criteria to consider suitable candidates as reference miRNAs for the qPCR data normalization: (I) the putative reference miRNA must be expressed in all samples; (II) the geometric mean of the C_t_ value of the miRNA must be lower than 35; and (III) no evidence for differential expression between the MEN1 group vs. the HC group.

To calculate target miRNA expression normalized with respect to the reference miRNA, the comparative C_t_ (ΔΔC_t_) method was used [75] with fold change = 2^−[(Ct miRNA of interest − Ct reference miRNA) MEN1 sample − (Ct miRNA of interest − Ct reference miRNA) control sample]^, where control sample represents the average C_t_ for the miRNA target minus average C_t_ for the reference miRNA in the control group [76].

### 4.6. Statistical Analysis

Data were expressed as mean ± standard deviation (SD) for categorical variables and as mean values ± standard error (SE) for miRNA expression levels. An χ^2^ test was used for categorical variables. The distribution of continuous data was determined using Shapiro–Wilk and Kolmogorov–Smirnov tests. Unpaired *t*-test with Welch’s correction or the two-tailed Mann–Whitney U test followed by Bonferroni multiple-comparison adjustment was used to identify significant differences in target miRNA expression between the MEN1 group and HC group, as appropriate. An ROUT outlier test was run to detect outliers. Receiver Operating Characteristic (ROC) curve analysis was carried out to evaluate the diagnostic value of c-miRNA in discriminating between MEN1 patients and HCs. Finally, assessment of the associated area under the ROC curve (AUC) at the 95% confidence interval (CI), as well as the best cut-off with the highest diagnostic specificity and sensitivity of the analyzed serum miRNAs, were determined and presented. *p*-values < 0.05 were considered statistically significant for all tests. Data were analyzed using GraphPad Prism software version 9 (GraphPad, San Diego, CA, USA) for Windows.

## Figures and Tables

**Table 1:** Comparison of demographic data between the 25 MEN1 patients and the 25 healthy controls.

	MEN1 Group	HC Group	*p*-Value
N	25	25	
Gender			0.23
Males	8	12	
Females	17	13	
Age			0.312
Mean	44.4	40.1	
±SD	12.12	10.25	

**Table 2:** Demographic data and clinical features of the 25 MEN1 patients.

Patient ID	Age (Years)	Gender	Inherited MEN1 Syndrome	Type of MEN1 Mutation	Age of Onset	1st Clinical Sign of MEN1	MEN1 Phenotype
1	50	F	Yes	Frameshift	39	PHPT	PHPT
2	54	F	Yes	Missense	28	Nephrolithiasis	PHPT
3	52	F	Yes	Nonsense	35	Insulinoma	PHPT, insulinoma, PRLoma, meningioma, cutaneous lesions
4	47	F	Yes	Frameshift	19	PRLoma	PRLoma, PHPT, insulinoma
5	46	F	Yes	Frameshift	32	PRLoma	PRLoma, non-functioning NET, adrenal hyperlasia
6	39	F	Yes	Missense	33	PHPT	PHPT
7	56	M	Yes	Missense	37	Nephrolithiasis	PHPT, non-functioning NET, LI, cutaneous lesions
8	38	M	Yes	Missense	31	PHPT	PHPT, PRLoma, cutaneous lesions
9	54	F	Yes	Splicing	49	PHPT	PHPT, PA, gastrinoma, lung carcinoid, LI
10	52	M	Yes	Frameshift	29	Nephrolithiasis, PHPT	PHPT, gastrinoma, lipoma, cutaneous lesions
11	20	F	Yes	Splicing	19	PHPT	PHPT
12	14	M	Yes	Frameshift	/	Asymptomatic	-
13	44	F	Yes	Splicing	38	Gastrointestinal disorders	PHPT, PA, gastrinoma, lung carcinoid
14	65	F	Yes	Frameshift	18	Nephrolithiasis	PHPT, PRLoma, non-functioning NET
15	33	M	Yes	Nonsense	15	PHPT	PHPT
16	47	M	Yes	Nonsense	40	Hypoglycemia	PHPT, insulinoma
17	36	F	Yes	Frameshift	30	PHPT	PHPT, PRLoma, insulinoma
18	35	M	Yes	Missense	15	Renal colic	PHPT, LI, cutaneous lesions
19	42	M	Yes	Missense	39	Lipoma of the gluteus	PHPT, non-functioning NET, LI, cutaneous lesions
20	48	F	Yes	Genetic variant in the 5’UTR	23	Hyperprolactinemia	PHPT, PRLoma, gastrinoma
21	45	F	Yes	Missense	22	PHPT	PHPT, PRLoma, non-functioning NET
22	37	F	No	Splicing	25	Hypoglycemic seizures	PHPT, PRLoma, insulinoma
23	41	F	Yes	Missense	15	PHPT	PHPT, PRLoma, insulinoma
24	70	F	No	Missense	58	PHPT	PHPT, PRLoma, insulinoma, gastrinoma, lung carcinoid, LI
25	46	F	Yes	Frameshift	20	Hypoglycemia	PHPT, PRLoma, insulinoma, gastrinoma, lung carcinoid, LI

M = male; F = female; PHPT = primary hyperparathyroidism; NET = neuroendocrine tumor; PA = pituitary adenoma; PRLoma = prolactin-secreting adenoma; LI = lipoma.

**Table 3:** Descriptive statistical values of Ct of the three putative reference miRNAs in 47 tested samples.

miRNA	Min	Max	Mean	SE
hsa-miR-23a-3p	22.5	33.3	27.23	0.40
hsa-miR-93-5p	25.47	40	29.99	0.66
hsa-miR-191-5p	20.10	40	29.7	0.82

**Table 4:** miRNAs used in the study.

miRBase ID	Role in the Study	Mature miRNA Sequence	miScript Primer Assay	miRbase Accession Number
cel-miR-39-3p	Exogenous spike-in miRNA to evaluate efficiency of RNA extraction and cDNA reverse transcription	UCACCGGGUGUAAAUCAGCUUG	219610	MIMAT0000010
hsa-miR-451a	Hemolysis indicator	AAACCGUUACCAUUACUGAGUU	MS0004242	MIMAT0001631
hsa-miR-23a-3p	Reference miRNA	AUCACAUUGCCAGGGAUUUCC	MS00031633	MIMAT0000078
hsa-miR-93-5p	Reference miRNA	CAAAGUGCUGUUCGUGCAGGUAG	MS00003346	MIMAT0000093
hsa-miR-191-5p	Reference miRNA	CAACGGAAUCCCAAAAGCAGCUG	MS00003682	MIMAT0000440
hsa-miR-24-3p	Tested c-miRNA	UGGCUCAGUUCAGCAGGAACAG	MS00006552	MIMAT0000080
hsa-miR-1301-3p	Tested c-miRNA	UUGCAGCUGCCUGGGAGUGACUUC	MS00031381	MIMAT0005797

## Data Availability

The data presented in this study are available on request from the corresponding author.
